# Performance and Robustness Testing of a Non-Invasive Mapping System for Ventricular Arrhythmias

**DOI:** 10.3389/fphys.2022.870435

**Published:** 2022-04-26

**Authors:** Krista Lesina, Tamas Szili-Torok, Emile Peters, André de Wit, Sip A. Wijchers, Rohit E. Bhagwandien, Sing-Chien Yap, Alexander Hirsch, Mark G. Hoogendijk

**Affiliations:** ^1^ Department of Cardiology, Erasmus MC, University Medical Center Rotterdam, Rotterdam, Netherlands; ^2^ Department of Radiology and Nuclear Medicine, Erasmus MC, University Medical Center Rotterdam, Rotterdam, Netherlands

**Keywords:** premature ventricular complex, ventricular tachycardia, non-invasive mapping, electrocardiography, ventricular arrythmia

## Abstract

**Background:** The clinical value of non-invasive mapping system depends on its accuracy under common variations of the inputs. The View Into Ventricular Onset (VIVO) system matches simulated QRS complexes of a patient-specific anatomical model with a 12-lead ECG to estimate the origin of ventricular arrhythmias. We aim to test the performance of the VIVO system and its sensitivity to changes in the anatomical model, time marker placement to demarcate the QRS complex and body position.

**Methods:** Non-invasive activation maps of idiopathic premature ventricular complexes (PVCs) using a patient-specific or generic anatomical model were matched with the location during electrophysiological studies. Activation maps were analyzed before and after systematically changing the time marker placement. Morphologically identical PVCs recorded in supine and sitting position were compared in a subgroup.

**Results:** Non-invasive activation maps of 48 patients (age 51 ± 14 years, 28 female) were analyzed. The origin of the PVCs as determined by VIVO system matched with the clinical localization in 36/48 (75%) patients. Mismatches were more common for PVCs of left than right ventricular origin [11/27 (41%) vs. 1/21 (5%) of cases, *p* < 0.01]. The first 32 cases were analyzed for robustness testing of the VIVO system. Changing the patient-specific vs. the generic anatomical model reduced the accuracy from 23/32 (72%) to 15/32 (47%), *p* < 0.05. Time marker placement in the QRS complex (delayed onset or advanced end marker) or in the ST-segment (delaying the QRS complex end marker) resulted in progressive shifts in origins of PVCs. Altered body positions did not change the predicted origin of PVCs in most patients [clinically unchanged 11/15 (73%)].

**Conclusion:** VIVO activation mapping is sensitive to changes in the anatomical model and time marker placement but less to altered body position.

## Introduction

The outcome, duration and risks of ablation procedures for premature ventricular complexes (PVCs) depend on their location of origin (Latchamsetty et al., 2015). To select the best strategic approach during ablation procedures, ECG algorithms can be used to estimate the origin of PVCs (Ouyang et al., 2002; Betensky et al., 2011; Al’Aref et al., 2015). Unfortunately, the positive predictive values of these ECG algorithms are suboptimal which is likely caused by the lack of correction for anatomical variations between patients in these algorithms (Gabriels et al., 2021).

Non-invasive mapping systems that do correct for anatomical variations between patients are available to localize the origin of PVCs (Cakulev et al., 2013; Misra et al., 2018). Some of these systems require high resolution body surface measurements recorded with electrode vests as an input (Cakulev et al., 2013). In contrast, the View Into Ventricular Onset system (VIVO, Catheter Precision, NJ, United States) uses ventricular arrhythmias recorded on a 12-lead ECG as an input. These recorded ventricular arrhythmias are then matched with forward simulated QRS complexes of a patient-specific anatomical model of the ventricles and torso to estimate the origin of focal ventricular arrhythmia (Misra et al., 2018). The different methods of the VIVO system offers several advantages. Firstly, the use of forward simulated QRS complexes allows for depiction of both the endocardial and epicardial surface of the ventricles. Secondly, the electrocardiographic input theoretically allows for the use of 12-channel Holter recordings as an input in patients with infrequent arrhythmias.

So far, the data on the performance of the VIVO system are limited. In a porcine model, ventricular stimulation sites were located with an average error of 18 mm (Oosterhoff et al., 2016). In an early validation study, the VIVO system was able to differentiate right from left ventricular origin in 20/22 arrhythmias of patients undergoing ablation procedures (Misra et al., 2018). The clinical value of non-invasive mapping system depends not only on the accuracy but also on its reliability under common variations of the inputs. The aim of this study was to test the performance of the VIVO system in clinical practice and to perform a robustness test by testing its sensitivity to changes in the anatomical model, time marker placement of the QRS complex and body position.

## Methods

We retrospectively analyzed the origin of ventricular arrhythmias as indicated by the VIVO system of patients undergoing catheter ablation for idiopathic PVCs and/or idiopathic ventricular tachycardias in the Erasmus MC, Rotterdam, Netherlands, from February 2020 to November 2021. The study was approved by the institutional review board of the Erasmus MC. The work had been carried out in accordance with the Declaration of Helsinki as revised in 2013.

### Non-Invasive Mapping Using the View Into Ventricular Onset System

The methods used for non-invasive imaging by VIVO have been described before (van Dam et al., 2009; van Dam et al., 2014; Misra et al., 2018). In short, the DICOM images of a contrast enhanced computed tomography scan (CT) or magnetic resonance imaging (MRI) of the thorax that was made in the outpatient setting were imported in the VIVO system. A patient-specific model of the ventricles and torso was created via a semi-automated process based on the molding of one of the triangulated reference models to the imported images. Next, a three-dimensional photograph was taken of the thorax of the patient in supine position after placement of a 12-lead ECG in the operation room immediately prior to the electrophysiological study. This three-dimensional photograph was then aligned with the patient-specific model of ventricles and torso upon which the exact locations of the ECG electrodes were indicated. A recording of a PVC of interest on the 12-channel ECG using the same electrode positions was then imported into the system. These PVCs were marked according to the recommendations by manually placing a marker after the T wave of the PVC on an isoelectric segment, a marker at the end and at the start of the QRS complex. The VIVO system then determined the most likely origin of the PVC by correlating the marked QRS complex with simulated QRS complexes from every cross point or node of the triangulated ventricular model. For this, an activation pattern was simulated from each cross point or node of the ventricular model by the calculation of activation isochrones. These simulated activation isochrones were translated to ECG waveforms which were matched in steps of 1 ms with the recorded ECG (van Dam et al., 2009; van Dam et al., 2013; van Dam et al., 2014).

### Electrophysiological Study and Catheter Ablation

**FIGURE 1:**
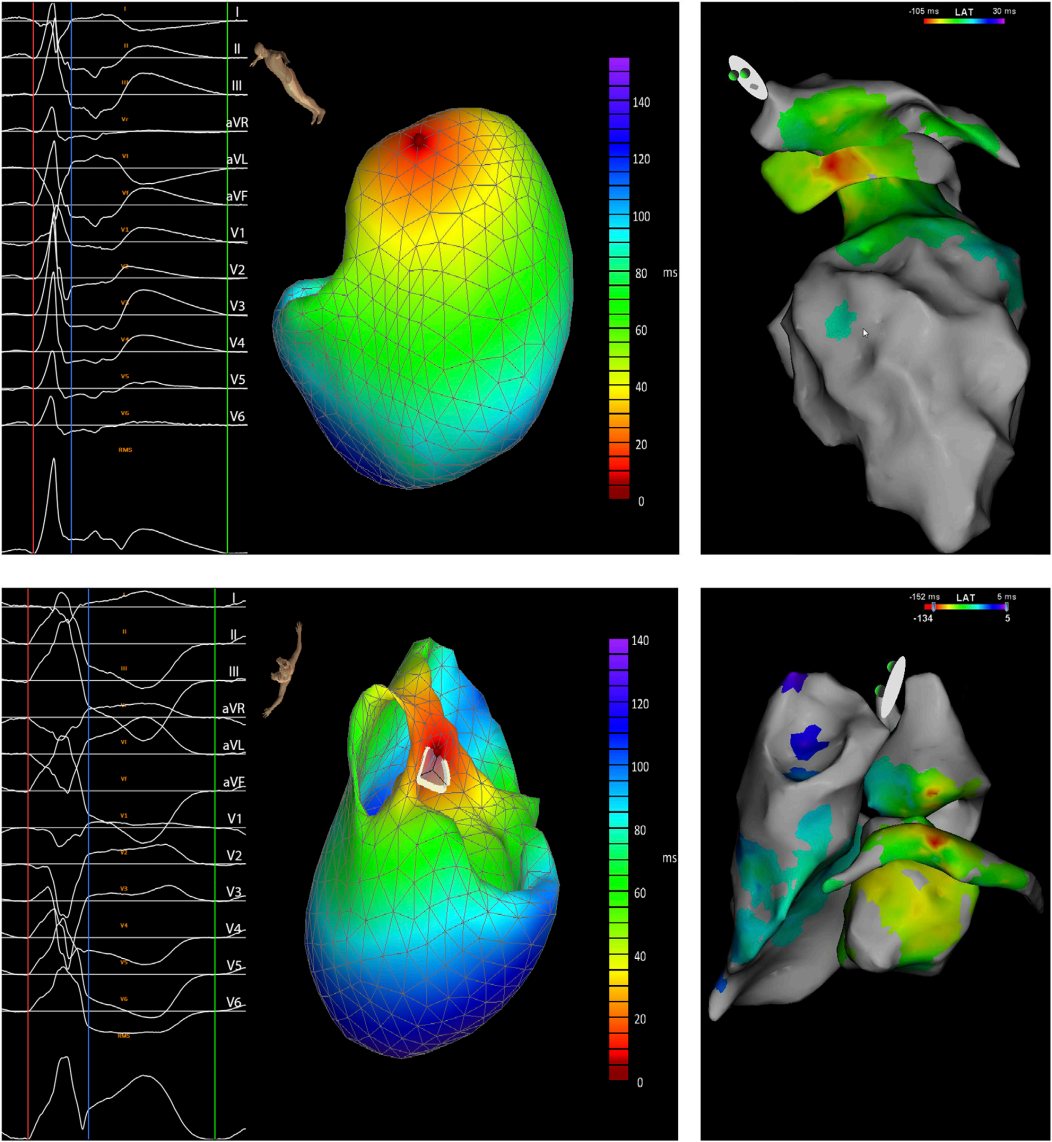
Two examples of predicted locations of a premature ventricular complex (PVC) by the VIVO system showing the analyzed PVC with the onset of the QRS complex marked by a red and the end by a blue line and triangulated heart model (left) and the electro-anatomical map with the local ventricular activation time (LAT) of the morphologically same PVC (right). The example at the top shows a good correlation with the ventricular activation measured from the great cardiac vein preceding the activation measured the left atrial appendage and ventricle. The PVC was successfully treated with radio-frequent energy applications from the left ventricle and great cardiac vein (patient 22). The example at the bottom shows the absence of a good correlation between the VIVO map (predicted location of origin being the right coronary cusp) and the electro-anatomical map with the ventricular activation recorded from the great cardiac vein preceding the activation measured from the right and left ventricle and coronary cusps. Ablation from great cardiac vein and left ventricle suppressed the PVCs but applications from the left coronary cusp were ineffective. After the procedure, the remaining PVCs disappeared and have not recurred during follow-up (patient 46).

All electrophysiological studies and catheter ablations were performed under local anesthesia with 1% lidocaine. Midazolam and fentanyl were added on indication. After gaining vascular access, heparin was administered following local protocol. Electro-anatomical mapping of the PVC was performed using a Navistar RMT Thermocool catheter in combination with CARTO3 (Biosense Webster, CA, United States) and the Niobe magnetic navigation system (Stereotaxis, St. Louis, MO, United States). Right ventricular mapping was performed via venous access. Left ventricular mapping was performed via retrograde aortic or intracardiac echocardiography guided transseptal access depending upon the discretion of the treating physician. Mapping of the epicardium was performed via the coronary sinus and great cardiac vein. The origin of PVCs was determined by semi-automated activation mapping in combination with the analysis of the bipolar and unipolar electrograms (Figure 1). Acute success of ablation was evaluated during a waiting time after the last application of typically 30 min and categorized as successful, uncertain or unsuccessful. Successful catheter ablation was defined as the absence of the targeted PVC during the waiting time. The success was considered uncertain in case the PVC burden was already very low before catheter ablation or if the PVC burden was markedly reduced after ablation and it was unclear whether the remaining rare PVCs should be considered as an effect of the ablation itself.

### Performance and Robustness Testing of the View Into Ventricular Onset System

**FIGURE 2:**
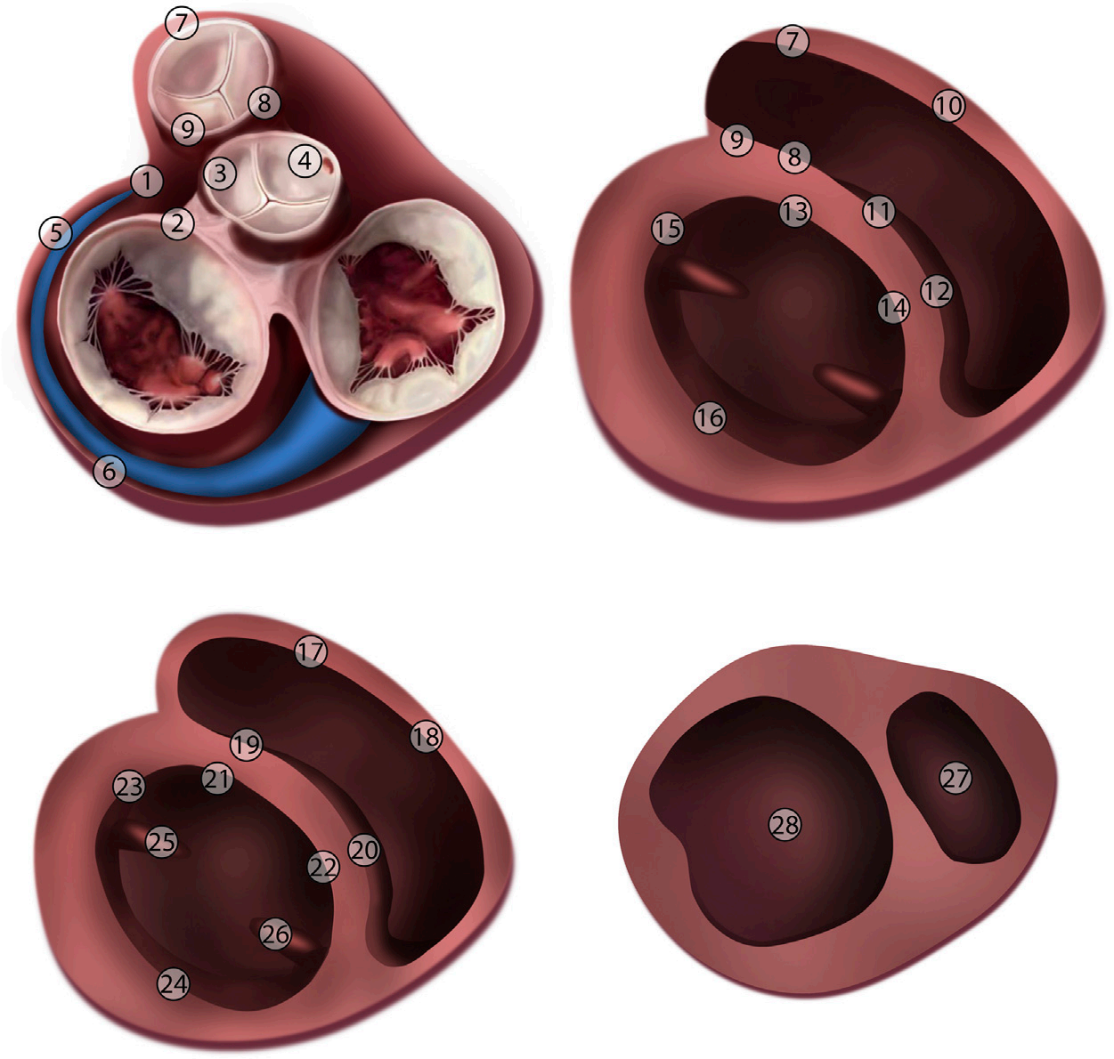
Ventricular heart model with 28 segments with epicardial, basal, mid and apical view. 1) Left ventricle summit; 2) Aortomitral continuity; 3) Left coronary cups; 4) Right coronary cusp; 5) Left ventricle anterolateral wall; 6) Left ventricle posterolateral wall; 7) Right ventricle outflow tract free wall; 8) Right ventricle outflow tract posteroseptal wall; 9) Right ventricle anteroseptal wall; 10) Right ventricle free wall; 11) Right ventricle anteroseptal wall; 12) Right ventricle posteroseptal wall; 13) Left ventricle anteroseptal wall; 14) Left ventricle posteroseptal wall; 15) Left ventricle anterolateral wall; 16) Left ventricle posterolateral wall; 17) Right ventricle anterolateral free wall; 18) Right ventricle posterolateral free wall; 19) Right ventricle anteroseptal wall; 20) Right ventricle posteroseptal wall; 21) Left ventricle anteroseptal wall; 22) Left ventricle posteroseptal wall; 23) Left ventricle anterolateral wall; 24) Left ventricle posterolateral wall; 25) Anterolateral papillary muscle; 26) Posteromedial papillary muscle; 27) Right ventricle apex; 28) Left ventricle apex.

We analyzed the performance by comparing the location of the PVCs according to the VIVO system with the earliest activation during the electrophysiological study using a novel 28-segment model of the ventricles focused on the most common locations of idiopathic PVCs (Figure 2). Both the same and a neighboring localization in the same cavity were considered a clinically relevant match between the VIVO system and electro-anatomical mapping.

We performed a more extensive analysis in the first 32 patients to determine the sensitivity of the VIVO system to variations in the anatomical and ECG input. To test the importance of the anatomical model, we compared the localization of a PVC using the patient-specific anatomical model with the localization of the same PVC using the anatomical model of one of the other patients (generic model) using the same 28-segment model of the ventricles (Figure 2). Both the same and a neighboring localization in the same cavity were considered a clinically relevant match between the VIVO system and electro-anatomical mapping. To analyze the importance of the correct ECG input, we varied the time marker placement and ran the VIVO system using the patient-specific anatomical model. We compared the origin of PVCs according to the VIVO system using the recommended time marker placement with the origin after systematically altering the onset or end of the QRS complex. The timing of either the onset or end of the QRS complex was altered from the recommended time marker placement in steps of 5 ms from −20 to +20 ms. Activation shifts within the same triangulated ventricular model were quantified by counting the minimal number triangle sides connecting the locations of origin of the PVC. In our analysis, we used either shifts in origins of PVCs of ≤2 triangle sides or of ≤4 triangle sides as clinically unchanged. The role of body position on mapping by the VIVO system was studied in a smaller subgroup of patients. We compared the origin of a PVC recorded in supine position with a morphologically identical PVC recorded in sitting position using the patient-specific anatomical model. As before, the activation shifts within the same triangulated ventricular model were quantified by counting the minimal number triangle sides connecting locations of origin of the PVC.

### Statistical Analysis

Data are depicted as mean ± standard deviation unless specified otherwise. A Chi-Square test was used to compare categorical variables. Comparison was considered significant in case the *p-*value was <0.05.

## Results

In total 48 patients with idiopathic PVCs and/or VTs were analyzed in this study. The age of the subjects was 51 ± 14 years, 28 patients were female and 20 were male. Forty-four patients underwent an ablation procedure for the first time whereas four underwent a redo procedure. The dominant idiopathic ventricular arrhythmias were PVCs in 43 patients and VTs in five patients. Eight of the patients had a reduced left ventricular ejection fraction defined as <45%. Of these eight patients, six underwent a contrast enhanced MRI before the procedure which did not show late gadolinium enhancement beyond the occasional enhancement at the right ventricular insertion point and that was the location of origin of the PVCs. The PVC burden during the preprocedural Holter ECG monitoring was 19.1 ± 13.9%. Multifocal PVCs, defined as <80% of the PVCs during Holter monitoring being monomorphic, were observed in 11 patients. The anatomical model required for the non-invasive mapping by the VIVO system was based on a contrast enhanced CT scan in 21 patients and on MRI in 27 patients. During the electrophysiological studies, the ventricular arrhythmias were determined to originate from the right ventricle in 21 patients with the most common location being the right ventricular outflow tract (*n* = 18). In the remaining 27 patients the ventricular arrhythmias originated from the left ventricle with the most common location being epicardial with the earliest activation in the coronary sinus, great cardiac vein or their side branches (*n* = 11). Acute catheter ablation success was reached in 34/48 (71%) and the outcome was considered uncertain in 4/48 (8%) of patients. No ablation was performed in 3/48 (6%) patients because ablation would present an unacceptable risk-benefit ratio due to the proximity of the bundle of His (*n* = 1), the imperfect timing in a side branch of the coronary sinus (*n* = 1) or a low PVC burden and multifocal PVCs after localization of the dominant morphology at the posteromedial papillary muscle. Baseline patient and procedural characteristics are summarized in Table 1.

**TABLE 1:** Patient and procedural characteristics.

	
Mean age (yr)	51 ± 14
Female/male	28/20
Mean BMI (kg/m^2^)	26.6 ± 4.0
LVEF <45%	8/48
PVCs mean burden on Holter before CA (%)	19.1 ± 13.9
Dominant arrhythmia (PVCs/VT)	43/5
Multifocal PVCs	11
First/Redo CA	44/4
Location PVCs (EPS)	
Right ventricle (*n* = 21)	
RVOT	18
RV moderator band	1
Parahisian	1
RV tricuspid annulus	1
Left ventricle (*n* = 27)	
LVOT	2
Aortic cusps	5
Aortomitral continuity	2
Epicardial	11
Papillary muscle	6
Posteroseptal	1
Acute CA success (Yes/Uncertain/No/No ablation)	34/4/8/3

BMI, body mass index; LVEF, left ventricle ejection fraction; PVC, premature ventricular complex; CA, catheter ablation; VT, idiopathic ventricular tachycardia; Multifocal defined as <80% of the PVCs during Holter monitoring being monomorphic; EPS, electrophysiological study; Mean ± standard deviation.

### Performance and Robustness Testing of the View Into Ventricular Onset System

The predicted locations by VIVO system using the patient-specific anatomical model and electrophysiological study matched clinically in 36/48 (75%) of patients. Mismatches were more common for PVCs of left than right ventricular origin [11/27 (41%) vs. 1/21 (5%) of cases, *p* < 0.01]. The PVCs with a clinical mismatch were identified during the electrophysiological study to originate from the moderator band (not incorporated in the patient-specific anatomical model), left ventricular summit (*n* = 4), right coronary cusp, left coronary cusp (*n* = 2), left ventricular anterolateral and posterolateral epicardium, left ventricular anteroseptal and posteroseptal wall (Table 2).

**TABLE 2:** Procedural characteristics and matching VIVO maps with invasive localization.

Right Ventricle						
Patient ID	Arrhythmia type	LVEF <45%	Location EPS	Location VIVO	Clinical match	Procedural outcome
1	PVC	No	Moderator band	16	No	Uncertain
2	PVC	No	7	7	Yes	Successful
3	VT/PVC	No	9	9	Yes	Successful
12	VT/PVC	No	9	9	Yes	Successful
13	PVC	No	9	9	Yes	Uncertain
15	PVC	No	7	10	Yes	Successful
19	PVC	No	9	9	Yes	Successful
21	PVC	No	7	9	Yes	Successful
23	PVC	No	9	7	Yes	Successful
26	PVC	No	9	9	Yes	Successful
28	VT/PVC	No	7	9	Yes	Successful
30	VT/PVC	No	9	9	Yes	Successful
32	PVC	No	8	9	Yes	Successful
33	PVC	No	9	9	Yes	Successful
34	PVC	No	19	8	Yes	No ablation
35	PVC	No	7	7	Yes	Successful
41	VT/PVC	No	9	9	Yes	Successful
43	PVC	No	11	11	Yes	Successful
45	PVC	No	9	9	Yes	Successful
47	PVC	No	9	9	Yes	Successful
48	PVC	No	8	7	Yes	Unsuccessful
**Left Ventricle**						
4	PVC	No	1	9	No	Unsuccesful
5	PVC	Yes	13	13	Yes	Successful
6	PVC	Yes	1	9	No	Unsuccesful
7	PVC	No	5	1	Yes	Unsuccesful
8	PVC	Yes	2	2	Yes	Successful
9	PVC	Yes	3	9	No	Successful
10	PVC	No	3	13	Yes	Successful
11	PVC	No	25	16	Yes	Unsuccesful
14	PVC	No	26	26	Yes	Successful
16	PVC	No	14	12	No	Unsuccesful
17	PVC	No	13	26	No	Successful
18	PVC	No	1	1	Yes	Successful
20	PVC	No	1	3	No	Successful
22	PVC	No	5	5	Yes	Successful
24	PVC	No	1	1	Yes	Successful
25	PVC	No	2	2	Yes	Successful
27	PVC	No	1	1	Yes	Uncertain
29	PVC	Yes	5	15	No	Unsuccesful
31	PVC	No	4	9	No	Uncertain
36	PVC	No	6	20	No	No ablation
37	PVC	Yes	26	26	Yes	No ablation
38	PVC	No	26	16	Yes	Unsuccesful
39	PVC	Yes	25	25	Yes	Successful
40	PVC	No	26	16	Yes	Successful
42	PVC	No	3	9	No	Successful
44	PVC	No	3	4	Yes	Successful
46	PVC	Yes	1	4	No	Uncertain

A clinical match was defined as the same and a neighboring localization in the same cavity. PVC, premature ventricular complex; VT, idiopathic ventricular tachycardia; LVEF, left ventricular ejection fraction; EPS, electrophysiological study.

The importance of the patient-specific anatomy in the VIVO system was tested by using the same PVC in the patient-specific or in the generic anatomical model of the ventricles and torso. A clinical match between the location identified during the electrophysiological studies and VIVO was more common using the patient-specific than generic model of the ventricles and torso (23/32 (72%) vs. 15/32 (47%) of cases, *p* < 0.05). Mismatches between the location identified during the electrophysiological studies and VIVO using the generic anatomical model were also more common at left than right ventricular locations of origin of the PVCs as determined during the electrophysiological study [15/19 (79%) vs. 2/13 (15%) of cases, *p* < 0.05].

**FIGURE 3:**
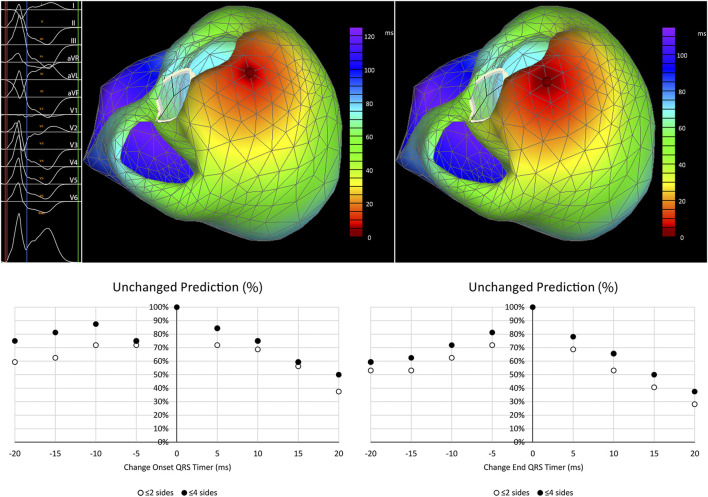
Shifts in the predicted origin of PVCs in relation to changes in time marker placement. Top left: ECG with time markers indicating onset (red), end of the QRS complex (blue) and an isoelectric point after the T wave (green). Top middle: Predicted activation map using standard timing placement. Top right: Predicted activation map using a delayed onset of the QRS complex (+10 ms) showing a clinically unimportant shift of one triangle side. Graphs below show the percentage of patients with clinically unchanged predictions of origin of the PVCs (defined as shifts ≤2 or ≤4 triangle sides and indicated as % of cases with an unchanged prediction) before and after altering the onset (left) and end marker (right) of the QRS-complex.

The origin of activation of the PVCs was compared between 32 activation maps made by VIVO with recommended and 512 maps with altered time marker placement. Subtle changes of 5 ms in the timing of either the onset or end of the QRS complex of the PVC resulted in shifts in the origin of activation in a clinically relevant number of patients (Figure 3). These shifts became more common in case the time markers were placed in the QRS complex (delayed onset or advanced end marker) or in the ST-segment (delaying the end marker of the QRS complex). We were not able to decipher a clear pattern in the direction of shifts in the origin of activation.

The dependence of the predicted origin of PVCs on body position was studied in a subgroup of 15 patients (patients 3–6, 18–26, 31, and 32). The change from supine to sitting body position did not change the predicted anatomical origin of PVCs in 11/15 (73%) when a change of ≤4 triangle sides and in 10/15 (67%) of patients when ≤2 triangle sides was considered clinically unchanged. No clear pattern could be discerned in the direction of the shifts in the cases in which altered body positions influenced the predicted location of origin.

## Discussion

In this study, we tested the sensitivity of the non-invasive ventricular arrhythmia localization by VIVO system to changes in the patient-specific anatomical model, the time marker placement and body position. Our data showed that the use of a patient-specific anatomical model is essential *in vivo*. The use of a specific anatomical model resulted in a better prediction of the location of the PVC than the use of a generic anatomical model. Secondly, our results illustrated the importance of the correct placement of the time markers to indicate the onset and the end of the QRS complex. Even modest alterations of the time marker placement could result in a clinically relevant shift in the origin of activation. Lastly, changing the body position from supine to sitting had less effect than we expected and resulted in a clinically relevant change in the predicted PVC localization in a minority of patients.

The importance of the use of a patient-specific anatomical model is in line with reports during the development of the VIVO system (van Dam et al., 2013; van Dam et al., 2014). The use of a patient-specific anatomy corrected the prediction in two patients with an inaccurate localization based on a generic model in early work on the VIVO system (van Dam et al., 2013). Furthermore, simulated misalignment of the location of the precordial ECG leads with the anatomical model has been shown to result in non-gradual and sometimes large shifts in the estimated location of ventricular arrhythmias (van Dam et al., 2014). Our results stress the importance of the use of a patient-specific anatomical model and the correct alignment of this model with the three-dimensional photograph. This alignment can be challenging in our experience along the vertical axis as anatomical features that can be used as a reference point for this, such as the base of the neck or the jugulum, are not always incorporated in the CT or MRI scans and, therefore, are not part of the patient-specific anatomical model. As a possible future solution, the sternal angle could be marked before taking the three-dimensional photograph and in the patient-specific anatomical model to help improve the alignment.

Despite our best efforts to create a well-suited patient-specific anatomical model and to align it closely to the three-dimensional photograph, the prediction of the VIVO system in our hands was not perfect. A clinical match with PVC location during the electrophysiological study was present in 75% of patients with left ventricular PVCs being more likely to result in a mismatch. A comparison of our results with previous work on the VIVO system is difficult based on the differences in definition of a clinical match. In the validation study of Misra et al., the location matched regionally in 11/13 (85%) mapped PVCs (Misra et al., 2018). We chose not to use a regional approach but to follow a clinical line of reasoning by considering only perfect matches and neighboring localization in the same cavity as a clinical match. A mismatch across the ventricular wall (f.e. from endocardial to epicardial) was not considered a clinical match in our scoring system because it would require a different approach of the ablation catheter. In case we would have used a regional approach, and neighboring locations across the ventricular wall would have been considered a match, our results would have been comparable with previous work with 44/48 (92%) of the locations of the VIVO system matching. The question remains what the expected optimal performance would be of a non-invasive mapping system like the VIVO system that uses the matching of forward simulated and recorded 12-channel ECGs. The closest clinical comparison to the VIVO system that may help us in answering this question would likely be pace mapping. In pace mapping studies, the morphology of paced QRS complexes using a roving catheter is compared with a clinical PVC based on the idea that pacing at the site of origin of the clinical PVC would result in the best match. In a study on the resolution of pace mapping in the right ventricular outflow tract, the area of pacing resulting in a good match was confined to 1.8 cm^2^ (Bogun et al., 2008). The resolution of pace mapping appears, however, to differ markedly between ventricular locations (Li et al., 2017). Thus, the expectation that the VIVO system would have a resolution and precision that can fully replace the need for activation mapping and the analysis of local electrograms during ablation procedures would appear over-optimistic and its accuracy may differ between ventricular locations. The location indicated by the VIVO system may, however, play a role in the procedural planning by influencing the order in which the different cardiac areas are mapped.

Beside these anatomical considerations, we also tested the sensitivity of the VIVO system to changes in time marker placement. Subtle changes in placement of the time markers could result in clinically relevant changes in the predicted origin of PVCs by the VIVO system by exclusion of (delayed onset or advanced end marker) or by incorporation of the discordant ST-segment deviation of the PVC (delayed end marker) in the QRS complex to be analyzed. Our data suggest that the reproducibility of non-invasive mapping systems based on forward modeling and matching of QRS complexes, such as the VIVO system, would benefit from a reproducible, and perhaps semi-automated, placement of the time markers. In addition, it may be helpful to show the calculated ECG waveforms considered to be the best match to the recorded ECG by the VIVO system. A visual confirmation of a good match between the two may enhance the sense of reliability of the estimated location of origin.

Lastly, we studied the role of body position on mapping by the VIVO system in a subgroup of patients. This has been of interest to us as a 12-lead Holter can also be used as an input for the VIVO system. The different body positions occurring during Holter monitoring may, however, influence the recorded ECG as it is known to influence the amplitude and axis of the QRS complex (Daralammouri et al., 2021). Interestingly, no clinically relevant difference was observed in the location of the PVCs indicated by VIVO between PVCs recorded in supine to sitting position in the majority of patients. The use of 12-channel Holter monitoring as ECG input for the VIVO system may, therefore, be feasible in patients with intermittent presence of symptomatic PVCs.

### Study Limitations

The number of patients in this single center study is limited and a selection of patients may have been included as the use of non-invasive activation mapping is not standard in our center. In addition, the study was performed in a retrospective and unblinded setting. Blinding the analysis process would have been difficult as the team performing the electrophysiological study was also involved in the analysis. We chose a study design for most of the analyses in which the influence of the operator is limited by using patients as their own control and we consider it unlikely that these factors will have had a material effect on our findings. As in any mode, assumptions are used in the VIVO system. Outspoken physiological changes resulting in slow conduction or anatomical abnormalities that influence the resistance in the thorax may influence its accuracy. The subgroup of patients used to study the effect of body position was small making the quantification of the effect less reliable. Lastly, in this retrospective study the work-up before non-invasive mapping and electrophysiological study was not standardized. We cannot fully exclude that a standardized work-up may have influenced the performance of the VIVO-system. The absence of standardization of the work-up will likely had less influence on the results of the robustness test in which patients served as their own control.

## Conclusion

Non-invasive activation mapping by VIVO is sensitive to changes in the anatomical model and time marker placement but less to altered body position. An automatic tool for time marker placement may help improve the reproducibility. Using 12-channel Holter monitoring as ECG input appears feasible.

## Data Availability

The raw data supporting the conclusion of this article will be made available by the authors, without undue reservation.
